# Implementation and utilisation of community-based mortality surveillance: a case study from Chad

**DOI:** 10.1186/1752-1505-6-11

**Published:** 2012-11-27

**Authors:** Sarah Bowden, Kai Braker, Francesco Checchi, Sidney Wong

**Affiliations:** 1 School of Public Health, Imperial College London, London, UK; 2 School of Medicine, Cardiff University, Cochrane Medical Education Centre, Heath Park, Cardiff, CF, 14 4YU, UK; 3 Médecins Sans Frontières (MSF), Am Köllnischen, Park 1, Berlin, 10179, Germany; 4 Faculty of Infectious and Tropical Diseases, London School of Hygiene and Tropical Medicine, Keppel St, London, UK; 5 Médecins Sans Frontières (MSF), 67–74 Saffron Hill, London, EC, 1N 8QX, UK

**Keywords:** Mortality, Surveillance, Death rate, Humanitarian, Conflict, Post-emergency, Chad, Refugees, Internally displaced persons (IDPs), Médecins sans frontières (MSF), Community health workers

## Abstract

**Background:**

Prospective surveillance is a recognised approach for measuring death rates in humanitarian emergencies. However, there is limited evidence on how such surveillance should optimally be implemented and on how data are actually used by agencies. This case study investigates the implementation and utilisation of mortality surveillance data by Médecins Sans Frontières (MSF) in eastern Chad. We aimed to describe and analyse the community-based mortality surveillance system, trends in mortality data and the utilisation of these data to guide MSF’s operational response.

**Methods:**

The case study included 5 MSF sites including 2 refugee camps and 3 camps for internally displaced persons (IDPs). Data were obtained through key informant interviews and systematic review of MSF operational reports from 2004–2008.

**Results:**

Mortality data were collected using community health workers (CHWs). Mortality generally decreased progressively. In Farchana and Breidjing refugee camps, crude death rates (CDR) decreased from 0.9 deaths per 10,000 person-days in 2004 to 0.2 in 2008 and from 0.7 to 0.1, respectively. In Gassire, Ade and Kerfi IDP camps, CDR decreased from 0.4 to 0.04, 0.3 to 0.04 and 1.0 to 0.3. Death rates among children under 5 years (U5DR) followed similar trends. CDR and U5DR crossed emergency thresholds in one site, Kerfi, where CDR rapidly rose to 2.1 and U5DR to 7.9 in July 2008 before rapidly decreasing to below emergency levels by September 2008.

**Discussion:**

Mortality data were used regularly to monitor population health status and on two occasions as a tool for advocacy. Lessons learned included the need for improved population estimates and standardized reporting procedures for improved data quality and dissemination; the importance of a simple and flexible model for data collection; and greater investment in supervising CHWs.

**Conclusions:**

This model of community based mortality surveillance can be adapted and used by humanitarian agencies working in complex settings. Humanitarian organisations should however endeavour to disseminate routinely collected mortality data and improve utilisation of data for operational planning and evaluation. Accurate population estimation continues to be a challenge, limiting the accuracy of mortality estimates.

## Background

Population death rates are a fundamental indicator of health status and monitoring mortality should be integral to every health system. However, in much of the world, deaths are not systematically recorded [1-3]; two-thirds of deaths go undocumented globally [2]. Death registration is particularly weak in complex emergencies where health and civil infrastructure are often poor and disrupted [4,5]. Mortality estimation should be a key part of any humanitarian health relief operation, as accurate mortality data are essential for monitoring trends in population health status, strategic planning, political advocacy and documentation of the impact of crises on human health [6,7]. *The Sphere Handbook* states that the crude death rate (CDR) is “the most useful health indicator to monitor and evaluate the severity of an emergency situation” [8]. The crude death rate is defined as “the rate of death in the entire population, including both sexes and all ages” [8]. Additionally, the *Sphere Handbook* notes that the under-5 death rate is more sensitive than CMR and is therefore an important age-specific indicator [8]. Mortality data can be collected prospectively through on-going surveillance or retrospectively through mortality surveys. Prospective surveillance is preferable because real-time collection allows for immediate analysis and timely reaction [6]. Furthermore, prospective surveillance theoretically features less bias than retrospective assessment [6]. Correct interpretation, dissemination and use of data are as important as data collection. Too often data remain unanalysed or there are long delays between data collection, analysis and subsequent publication [1,9]. There is an ethical obligation to the community to utilise any routinely collected data [10]. In addition, the dissemination of information across organisations and to communities themselves is frequently neglected [11]. Although mortality surveillance systems are widely used by humanitarian organisations, no known guidelines for the implementation of prospective mortality surveillance systems exist, and evidence on their effectiveness is scarce [12]. Furthermore, there is little published on the utilisation of mortality data for monitoring and decision-making by humanitarian agencies. Practical experience of implementation of surveillance should be shared to assist improvement of methodology. Evidence of utilisation of mortality data to improve the humanitarian response should be published in support of expert recommendations for ongoing implementation of mortality surveillance in complex emergencies.

In this case study we conducted key informant interviews and reviewed operational reports to describe and evaluate a community-based mortality surveillance system implemented over 4 years (2004–2008) in 5 sites by the humanitarian medical organisation, Médecins Sans Frontières (MSF) as part of a programme assisting refugee and internally displaced person (IDP) populations in eastern Chad. We describe trends in mortality data and how these data were used by MSF in planning and advocacy. Our aim is to share lessons learned to assist the further development of prospective mortality surveillance systems in conflict and complex emergency settings.

## Case description

### Methods

#### Ethical considerations

The study met the standards of the MSF Ethics Review Board for the retrospective analysis of routinely collected programmatic data and thus was exempted from formal review.

#### Study site and population

**Figure 1:**
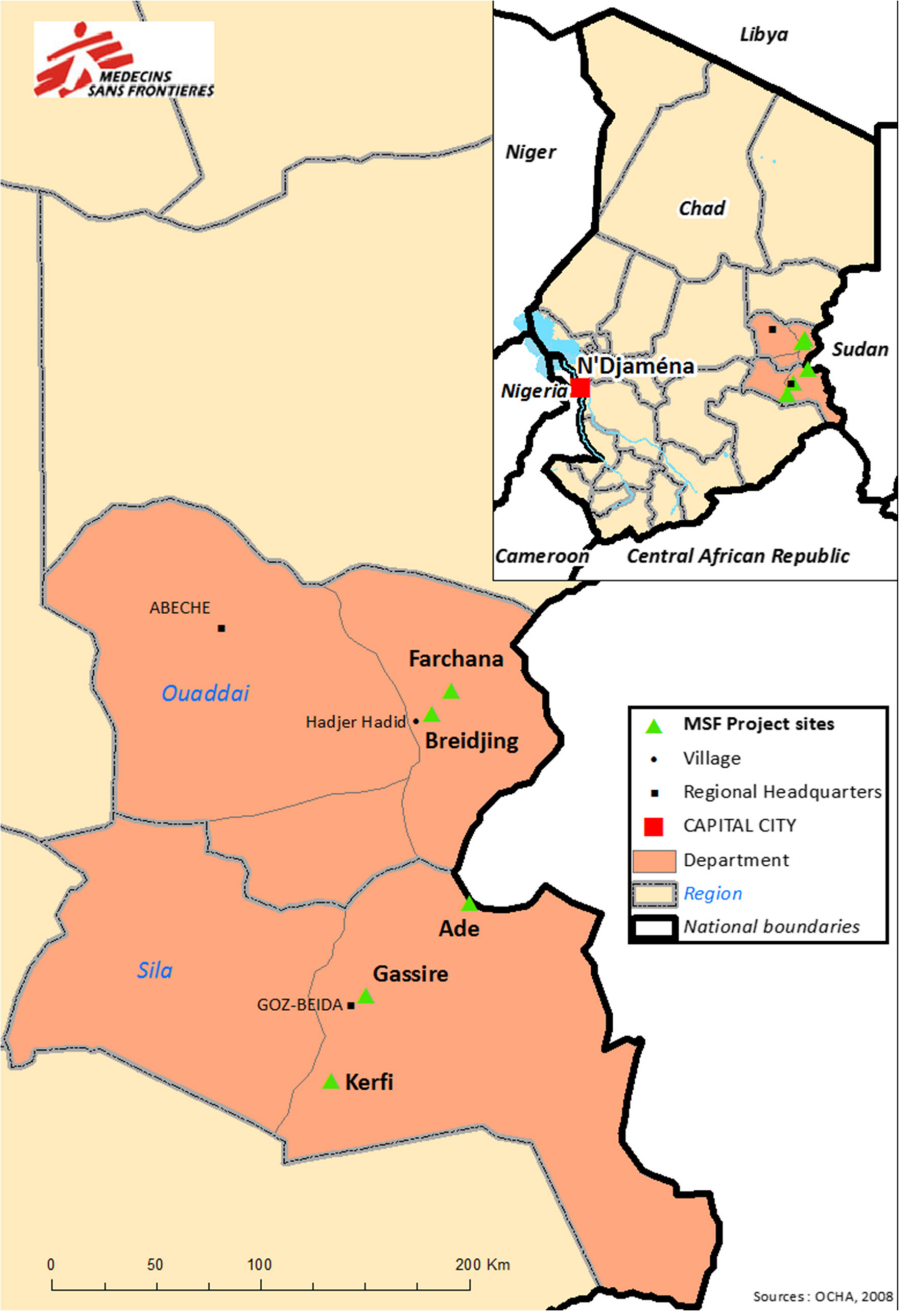
MSF project sites in eastern Chad.

Starting in July 2003, refugees from the conflict-affected region of Darfur fled to eastern Chad. By the end of 2008, the number of Sudanese refugees totalled 250,000 [13]. In 2006 and 2007, Sudanese Janjaweed militias carried out cross-border raids and exploited long-standing tensions between Chadian ethnic groups leading to widespread inter-ethnic violence. Armed conflict between the army and Chadian rebel groups also broke out. By the end of 2008, 180,000 Chadians were displaced from their homes [13] and many were living in IDP camps. Farchana and Breidjing camps in the Hadjer Hadid area assisted Sudanese refugees. Gassire, Kerfi and Ade camps in Goz Beida assisted Chadian IDPs (Figure 1). MSF estimates of the population of camps at the end of the period analysed were: Farchana- 20,000 (July 2008), Breidjing- 30,473 (July 2008), Gassire- 14,754 (December 2008), Ade- 15,000 (December 2008), Kerfi- 13,000 (December 2008).

#### MSF activities

**Figure 2:**
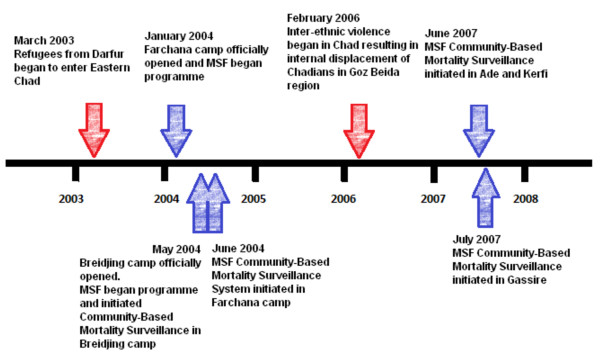
Timeline of initiation of mortality surveillance systems by MSF.

MSF began working with refugees in Farchana camp in January 2004 and in Breidjing camp in May 2004. Community health worker (CHW) mortality surveillance was implemented in May and June 2004 in Breidjing and Farchana camps respectively. MSF scaled up work with IDPs in the Goz Beida area in November 2006. CHW mortality surveillance systems were initiated in June 2007 in Ade and Kerfi programmes and July 2007 in Gassire (Figure 2). After 2008 medical care at Farchana and Breidjing was handed over to other NGOs. All five MSF projects included basic primary care outpatient and emergency triage clinics; referral systems for local hospitals; and nutrition, maternal health, mental health and sexual and gender-based violence programmes.

#### Data collection

**Figure 3:**
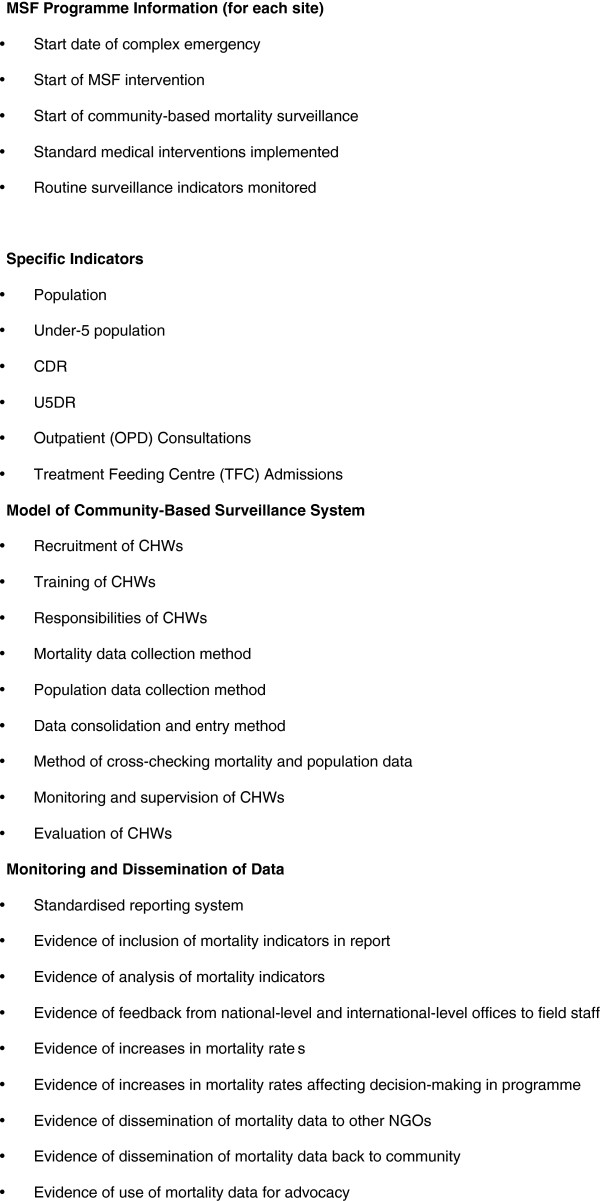
Data extraction tool.

**Figure 4:**
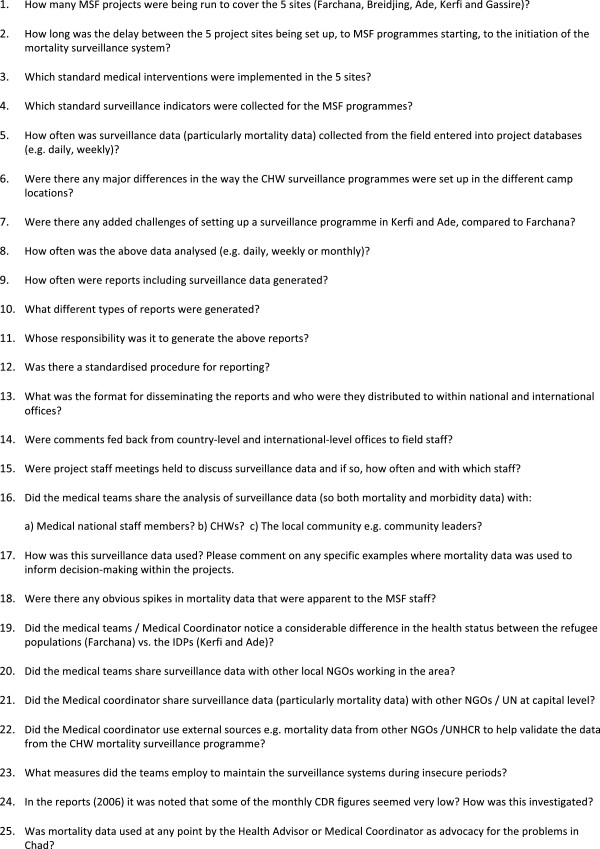
Standardised Questionnaire for Key Informants.

Standard criteria to measure performance of a surveillance system [14] were used to develop a data extraction tool to analyse MSF operational reports and a questionnaire for key informants (Figure 3 and 4). Operational reports at national and project level for 2004–2008 were identified. Reports included ‘monthly medical reports’ and ‘four-monthly reports’. 27 of 44 monthly medical reports and 14 of 15 four-monthly reports were available for analysis. Two key informants were interviewed: the Health Advisor for Chad from September 2004 to 2008 who was also interim Medical Coordinator for October 2004, and a medical staff member from 2006–2007 who also served as a programme medical coordinator in 2006. Key informants completed a standardised questionnaire (Figure 4), followed by a semi-structured interview.

## Results

### Implementation of the surveillance system

For each project site, a team of CHWs were recruited from the camp populations. Camps were mapped and separated into groups of around 1000 people. Two CHWs were assigned to each group and given a weekly report form (in Arabic) to record births and deaths. On interviewing households about deaths CHWs asked about the name, age, sex, symptoms before death and evidence of violence or an accident. Their location in the camp and whether a health post had been attended was also asked to prevent duplicates in data entry. CHWs had weekly supervision meetings with a supervisor. The number of deaths were collated weekly from CHWs by the supervisor and entered onto the on-site Excel medical database. Deaths reported as having occurred in the MSF health clinic were crosschecked with clinic records. The total number of deaths were compared with weekly graveyard counts undertaken by a graveyard-watcher. The graveyard-watcher interviewed families attending the burial site and collected data on name, age, sex, symptoms before death, evidence of violence/accidents, attendance of any health post and where they were based in camp. Where graveyard watchers reported deaths, CHWs would confirm with community leaders. These deaths were cross-checked with CHW records to identify duplicates and any deaths not present on the CHW reports were investigated by consulting with community leaders. Key informants noted that it was very difficult to get reliable information regarding symptoms before death, violence and accidents. The population figures used for the calculation of mortality rates included estimates collected by MSF CHWs and UNHCR registration records. However the source of population estimates was not consistently recorded with CDR figures in monthly reports.

### Trends in surveillance data

#### Refugee camps (Farchana and Breidjing)

**Figure 5:**
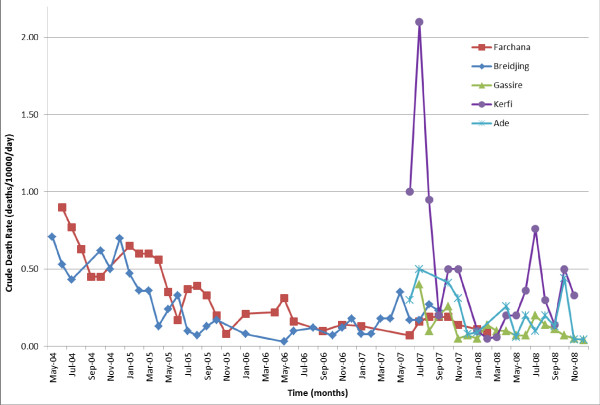
Crude death rates (CDR) in 5 MSF project sites in eastern Chad 2004–2008.

**Figure 6:**
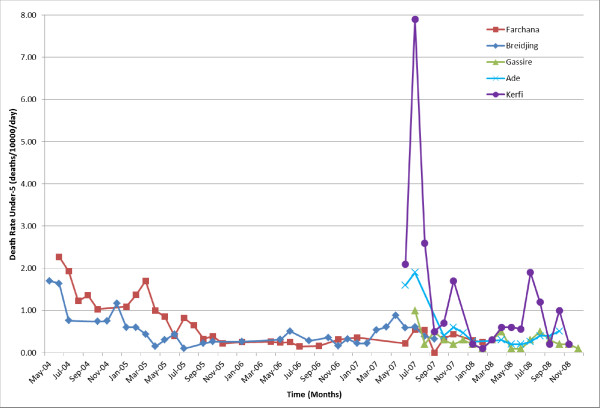
Under-5 death rates for 5 MSF project sites in eastern Chad 2004–2008.

In Farchana, the CDR was 0.9 deaths/10000/day in June 2004, and declined steadily to 0.2 by July 2008 (Figure 5). The CDR in Breidjing followed a similar pattern, falling steadily from 0.7 in March 2004 to 0.1 in January 2008. These trends were mirrored by under-5 crude death rates (U5DR; deaths in children younger than 5 years) (Figure 6).

#### IDP camps (Gassire, Ade and Kerfi)

In Gassire, the CDR was initially low and slowly declined from 0.4 in July 2007 to 0.04 in December 2008 (Figure 5). In Ade, the CDR fell from 0.3 in June 2007 to 0.04 in December 2008. Kerfi experienced the highest mortality, with an initial recorded CDR of 1.0 in June 2007 which rose to 2.1 in July 2007. CDR then rapidly decreased; the last available rate was 0.3 in November 2008. U5DR in Kerfi was 2.1 initially before climbing to 7.9, four times the emergency threshold suggested for children aged under 5 years and about eight times the typical rate in stable Sub-Saharan African settings [8]. The last available rate was 0.2 in November 2008 (Figure 5).

### Data utilisation

**Figure 7:**
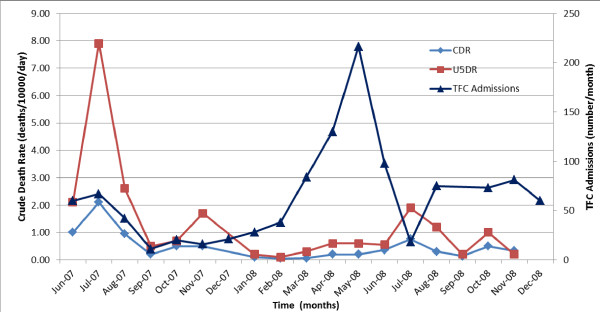
**Crude death rates and TFC admissions, Kerfi, 2007–2008.** TFC=therapeutic feeding centre.

Data collected from the community surveillance system were regularly monitored and reviewed by programme staff during weekly medical team meetings (Table 1). CDRs were included in MSF operational reports, which were distributed up the operational command chain on a monthly and four-monthly basis to the country management team and MSF headquarters, respectively. In November 2007 a standardized form for monthly reports was introduced, which improved the completeness of mortality data in monthly reports (Table 1). On two occasions, mortality data from Kerfi were used as an advocacy tool. In July 2007, the high CDR and U5DR, linked to high incidence of diarrhoeal disease and poor water quality, were used by MSF programme staff to lobby other organisations (International Committee of the Red Cross, Oxfam, UNHCR) to increase provision of drinking water and sanitation programmes. Secondly, elevated U5DR in July 2008 (Figure 7), with a preceding peak in admissions to the therapeutic feeding centre, led to an investigation of malnutrition in Kerfi camp. The findings were used to lobby the World Food Programme to review and improve food distribution in Kerfi camp. During the study period, there were no internal programme evaluations which used data from the community mortality surveillance system.

**Table 1:** Evidence of mortality data utilisation by MSF

**Activity**	**Entire period studied**	**Pre-introduction of form: 05–2004 to 11-2007**	**Post-introduction of form: 11–2007 to 12-2008**
**CDR discussed in medical team meetings**	Weekly	Weekly	Weekly
**CDR included in monthly report**	75% (n=36/48)	64% (n=21/33)	100% (n=15/15)
**Trends noted**	40% (n=19/48)	12% (n=4/33)	100% (n=15/15)
**Population figures included in monthly report**	46% (n=22/48)	21% (n=7/33)	100% (n=15/15)
**Used for advocacy**	2 occasions	1 occasion	1 occasion
**Used for programme evaluation**	No evidence	No evidence	No evidence

## Discussion and evaluation

### Lessons learned

Prospective monitoring of mortality in a complex emergency setting can be extremely difficult. This case study highlighted the challenges of mortality data collection along with the strengths and weaknesses of the MSF community-based surveillance system. Challenges faced and lessons learned are described here.

### Validity

Several retrospective mortality surveys were undertaken in 2006–2007 in the same area (Table 2). Surveys from Farchana and Breidjing showed an average CDR of 0.4 deaths/10000/day and U5DR of 0.6 for 2006–2007 [15], i.e. higher than the rates estimated by the MSF system, which at that time fluctuated around a CDR of 0.1-0.2 and U5DR of 0.2-0.5. Surveys in the Goz Beida area [15,16] in 2006–2007 also found mortality rates higher than MSF figures for Ade, Kerfi and Gassire IDP camps (MSF data only available from July 2007 onwards). We must be cautious when comparing survey to surveillance data. Time frames and camp characteristics may have differed, and surveys also carry considerable bias [17,18]. However, crude comparisons and broad ranges of baseline pre-conflict death rates in both Sudan and Chad suggest that the MSF surveillance system may have underestimated mortality. This underestimation may have occurred due to under-reporting of deaths, overestimation of the population denominator or a combination of both, and may have considerably confounded the interpretation and use of data, suggesting a more favourable picture than reality. Despite the possible underestimation of mortality by the surveillance system, it can be argued that surveillance allows monitoring of trends and therefore provides valuable information on any change in mortality rates [19]. Previous reports suggest that prospective surveillance in post-emergency camps can achieve reasonably high sensitivity [19]. In the Central African Republic, rural weekly surveillance accompanied by intense training and supervision detected >90% of deaths [20]. However, it is plausible to assume that the sensitivity of surveillance may decay unless intense supervision complemented by spot checks, refresher training and validation exercises are carried out; furthermore, communities that do not see a clear benefit for ongoing data collection may reduce their participation and increasingly under-report deaths. The MSF system stored and analysed data electronically, which reduces errors [7,14]. While data were partly validated by cross-checking with alternate sources (health facilities, graveyards), this might not have been sufficient to capture deaths that took place outside health facilities and people who were not buried in recognised sites. Multiple sources could have been used to estimate the proportion of deaths detected by the system by using capture-recapture techniques [21-23], but these require considerable statistical expertise.

**Table 2:** Comparison of MSF mortality surveillance data and mortality survey estimates available for Eastern Chad. 2006–2010

**Study**	**MSF mortality surveillance system**						**Retrospective mortality survey estimates**			
	**December 2006**		**July 2007**		**December 2007**		**2006-2007(Source: CRED, 2011) [**[15]**]**		**May 2007(Source: Guerrier et al., 2009) [**[16]**]**	
Indicator**	**CDR**	**U5DR**	**CDR**	**U5DR**	**CDR**	**U5DR**	**CDR**	**U5DR**	**CDR**	**U5DR**
**Farchana**	0.14*	0.31*	0.16	0.55	0.13	0.32	0.4	0.6		
**Breidjing**	0.18	0.33	0.17	0.61	0.31	0.32	0.4	0.6		
**Gassire**			0.4	1.0	0.07	0.30				
**Ade**			0.5	1.9	0.08	0.47				
**Kerfi**			2.1	7.9	0.5*	1.7*				
**Dogdore**							0.9	2.0		
**Goz Beida**									1.8	4.1

CDR = Crude death rate; U5DR = Death rate in under-5 population; *November figure taken when December figure unavailable; ** Deaths/10000/day.

Note: The survey undertaken by CRED [15] was of mortality in the Goz Beida region. This region is the location of MSF projects Gassire, Ade and Kerfi. The survey undertaken by *Guerrier et al.*[16] measured mortality in Dogdore, an IDP settlement located 30Km west of the Chad-Sudan border and 120Km east of Goz Beida (and MSF projects in Gassire, Ade and Kerfi).

Estimating population size in a complex emergency setting is often challenging due to weak infrastructure, security issues and mobility of the population [12]. Mortality estimation would have been greatly aided if the estimates of population generated by community health workers and UNHCR were validated by performing additional assessments. In addition more careful reporting of which source was used for the population estimate would have allowed clearer retrospective analysis. UNHCR population figures from Farchana and Breidjing camps for 2004 and 2005 [24,25] are available for comparison with population estimates in MSF operational reports (Table 3); in 2004, use of MSF population figures would have led to a moderate over-estimation of death rates in Farchana and a considerable under-estimation in Breidjing, assuming UNHCR figures to be closer to the truth. The highly dynamic populations in the MSF programmes rendered data collection challenging and could have led to inaccurate population estimations. Also, households may have inflated their numbers in order to receive more aid [14]. Population overestimation may explain the low mortality rates recorded by MSF compared to survey results. Conversely, UNHCR may also have been underestimating population size, for example by not including unregistered populations. The population figures for children under-5 were calculated by assuming that they comprised 20% of the overall population, a typical value for most of sub-Saharan Africa. However, a count of under-5s in Farchana found they comprised 28% of the population. Therefore the under-5 population may have been underestimated, and U5DR over-estimated. To improve the validity of the U5DR, representative rather than estimated under-5 population figures should be obtained for calculation.

**Table 3:** **MSF and UNHCR population estimations for Farchana and Breidjing at end of 2004 and 2005**[24,25]

**Refugee camp**				
	**MSF**	**UNHCR**[24]	**MSF**	**UNHCR**[25]
**Farchana**	16,250	19,070	17,485	17,250
**Breidjing**	38,100	29,280	27,500	26,770

### Simplicity and flexibility

A surveillance system should be simple and flexible [14]. CHWs collected data on a daily basis, which were collated weekly before being included in monthly reports for dissemination. The system was easy to supervise and monitor through weekly meetings with staff. The system was flexible to a highly evolving population where camp sizes changed and data collection methods had to adapt correspondingly. The flexibility of the surveillance system was also challenged by repeated security threats; there were 6 incidents across the 5 camps during the 4-year period where mortality data were not recorded for the month. Mortality surveillance data were recorded in Excel databases and entered into 4-monthly reports as well as in monthly medical reports. Therefore mortality data were recorded despite monthly medical reports not being produced. This improved the availability of mortality surveillance data both at the time and for retrospective analysis. Furthermore the surveillance model was flexible enough to be implemented in the same way in several different sites.

### Appropriateness

The use of CHWs recruited from the local refugee and IDP populations may have increased the acceptability of the system. Using local CHWs may help to overcome some of the social, political, economic and cultural barriers [19] to data collection on deaths. However, multiple language barriers between CHWs, CHW supervisors and expatriate staff were sometimes a challenge. Regular re-emphasis of data collection methods with CHWs was necessary.

### Timeliness of implementation

Community-based mortality surveillance systems were initiated in the MSF programmes after the beginning of the medical intervention. From programme initiation, mortality systems took 2, 2, 3, 4 and 5 months to initiate for Breidjing, Ade, Farchana, Kerfi and Gassire camps, respectively. This delay may have hidden initially higher mortality rates. Implementation of community-based surveillance is generally too slow in the emergency phase resulting in a lack of data for the period where mortality is highest and where rapid information is essential for allocation of resources. Initiating a well-designed surveillance system takes time. However, the importance of mortality surveillance for programme monitoring means that it should be recognised as an early priority and implemented immediately where feasible [26].

### Dissemination of data

Surveillance data were collected weekly and project team meetings were held weekly, allowing review of mortality data and the ability to react quickly to changes. However, as data passed up the chain, the regularity of reporting decreased. Medical reports were analysed monthly at national level and every four months at international level. The introduction of a standardised reporting format in November 2007 increased the frequency of inclusion of mortality data (by 36%), population denominators (79%) and trends in data (88%) in monthly medical reports. Data on morbidity, mortality, admissions in nutritional programs and vaccination were shared with UNHCR on a monthly basis. Surveillance data were also shared with other UN agencies, operational non-governmental organisations, the Ministry of Health and community leaders. Additional action to share data then occurred when mortality rates increased. Regular meetings were held with community leaders to discuss the health status of the communities. Further information on the regularity and extent of data-sharing was not available. Sharing of surveillance data between different actors in the complex emergency setting should be encouraged to prevent duplication in data collection, improve completeness of information and to share skills and resources [7].

### Other challenges

There were six incidents where death rates were not recorded in databases due to security threats and absences of the expatriate and national staff team. Security incidents also affected the production of monthly medical reports on several occasions. This reflects one of the major challenges the surveillance system faced and lack of data may have hidden important fluctuations due to violence or worsened access to medical, food and other aid programmes.

### Limitations

There were several limitations to this case study. Data were analysed retrospectively through operational reports and key informant interviews; study sites were not visited. Consequently information available for evaluation was limited by the availability and content of reports. Discontinuity due to security problems, staff evacuations and staff shortages were the main reason for missing monthly reports. Also some reports were no longer available at the point of analysis in 2011. Data in missing reports may have differed from that available and contained important information for this study. Furthermore, information available was limited by the recall of key informants; incorrect recall may have led to inaccuracies and bias. Costs are an important part of system evaluation. Prospective mortality surveillance is believed to be feasible and cost-beneficial in most humanitarian relief programmes [12]. Data were unfortunately not available to quantify the resource implications of this surveillance system.

## Conclusions

In the study sites, CDRs generally decreased between 2004 and 2008 and were below recognised emergency thresholds. There were two instances in Kerfi where death rates were seriously elevated; increased mortality highlighted the need for intervention and data were used by MSF to lobby other organisations for improved water quality and food distribution. This case study found that community-based mortality surveillance is useful for population health status monitoring and advocacy in the post-emergency phase. We therefore provide evidence to support the expert opinion that CDR and U5DR are key indicators in humanitarian response [8]. There is however, no known standardised method for community-based mortality surveillance in emergencies [1,12] and many challenges are faced in obtaining accurate mortality data in such settings. This case study provides lessons learned by MSF, which may be useful for organisations implementing mortality surveillance in similar settings. We also highlight the areas where further improvement is necessary for the production of accurate mortality data in complex emergencies. In this case study, mortality rates were seen to be declining and generally below emergency thresholds at the implementation of mortality surveillance. Implementation of community-based surveillance is often too slow in the emergency phase. Mortality surveillance should be recognised as an early priority in the initiation of humanitarian programmes and as a useful tool in both the emergency and post-emergency phase. The need for improved population estimates to improve the accuracy of mortality data cannot be underemphasised. A crucial element of any mortality surveillance system should be establishing the procedure or source for population estimation and ensuring accurate and up-to-date figures are used. Where possible, ongoing population estimation should be an integral part of the surveillance system. Furthermore, the under-5 population should be disaggregated as opposed to assuming the figure of 20% of total population. Estimating population size is challenging in complex emergencies where populations are often highly mobile, however the importance of good estimates for mortality surveillance should ensure it is prioritised and that resources are allocated to monitoring population numbers.

Where the magnitude of mortality rates determined by surveillance are thought to be inaccurate, most commonly underestimated, following trends in data can provide useful information on any change in status [19]. Where available, organisations should compare survey results to surveillance data, which may provide more reliable estimates of magnitude. To evaluate the sensitivity and specificity of the data there is a need to systematically carry out validation exercises, ideally through the employment of capture-recapture statistics. Possible alternative sources of mortality data for validation purposes have been indicated previously, and include health facilities, graveyard monitors, religious and civil leaders, and other community health workers [19,22]. A lack of standard reporting procedures is one of the main problems in recording mortality under emergency conditions [22]. In this case study, implementation of a standardised reporting form improved the frequency of inclusion of data in monthly reports. Standardised reporting procedures could improve recording of surveillance data and thus its availability for programme monitoring, planning, evaluation and operational research.

Data sharing between organisations continues to be a major problem in complex emergencies hindering the efficiency of the relief effort. Data should be promptly disseminated to other relief stakeholders and the community themselves both on a regular basis and when mortality rates rise. The procedures for data dissemination should be defined when designing the surveillance system along with the measures for data storage in order to ensure future availability. Monitoring CDRs in emergency settings provides an indication of the magnitude of the crisis and can be used to evaluate the overall impact of humanitarian programmes. The usefulness of CDRs in supporting planning of individual interventions within a relief programme is limited. To this end, they need to be used in conjunction with other indicators such as cause specific mortality, disease specific morbidity data and service coverage and utilisation data. Improved vital registration globally should be a long-term goal of the international community; however, this will require large technical and financial investment [27]. A feasible short-term goal is to ensure that international humanitarian organisations note the Sphere Project recommendations [8] and recognise mortality surveillance as a vital component of any programme [1].

## Abbreviations

MSF: Médecins Sans Frontières; IDPs: Internally Displaced Persons; CHWs: Community Health Workers; CDR: Crude Death Rate; U5DR: Under 5 Death Rate; UNHCR: United Nations High Commission for Refugees; UN: United Nations.

## Competing interests

The authors declare that they have no competing interests.

## Authors’ contributions

KB carried out data collection and conceived the study. SW participated in conception and design of the study, coordination, data analysis and interpretation, and drafting the manuscript. SB contributed to design of the study and participated in data analysis and interpretation and drafting of the manuscript. FC participated in study design, review and revision of the manuscript. All authors reviewed and approved the final manuscript.
